# Transfusion

**Published:** 1874-04

**Authors:** 


### Miscellaneous.

### Transfusion.

At a recent meeting of the New York Academy of Medicine, Dr Fordyce Barker, spoke as follows:

The notion of transfusion of blood from one individual to another was entertained by the ancients. Ovid, in his Metamorphoses, refers to Media, the sorceress, as having power to rejuvenate old age, restoring to youth and vigor, by two processes; namely, by injecting into the veins the juice of certain herbs, and by injecting into the veins the blood of a young person. Practically, however, this experiment, so far as known, was never attempted by the ancients.

In 1657 Sir Christopher Wren, having made some experiments, suggested the possibility of the operation, in a communication which he made to the Royal Society of London in the same year.

In 1665 Dr. Lower experimented by transfusion of blood from one animal to another, and at about the same time Denys, of France, working independently, commenced a series of experiments of like nature. To Denys belongs the honor of being the first to perform the operation upon the human subject, which was done in the year 1666, and was a success. The operation was performed upon a young person who was sick with fever, and had been bled, vomited, and purged twenty-two times. The surface of the body was cold, the patient was pulseless, respiration was extremely rapid, mind delirious, etc., when Denys proposed transfusion, by which means he succeeded in restoring his patient and complete recovery followed. His second case was one of very similar character, and occurred in the person of the son of a Minister Plenipotentiary to Paris, who had been bled in the treatment for a fever until extremely exsanguinated. Transfusion was performed, and a similar success followed the operation. The results of these two operations gave rise to the most extravagant enthusiasm. It was alleged that the insane could be cured in this manner, and that the old could be made young. In the course of one year, however, so many fatal results had taken place, that the operation was interdicted by law, except as it was permitted by the Faculty of Paris. If that body granted permission, the operation could be performed.

From that time until 1685 the operation fell into disuse in all parts of the world. In 1685 Prof. Harwood, of Cambridge, commenced a series of experiments upon animals. In one of his experiments, which was performed upon an old horse, the animal is said to have raised up and commenced feeding.

In connection with a communication made by him to the Royal

Society in England, a man by the name of Cogor consented to have the operation performed by taking the blood of a sheep, and it is reported that the man suffered no harm from the experiment. A second experiment was performed upon the same man, but no history of the result could be obtained. No attempt, however, was made to resort to this operation for the purpose of restoring life in desperate cases until 1819, when the experiment was revived by Dr. Blundell, of London. He commenced a series of experiments to determine what would be the results of transfusion of blood from a vigorous animal to one that was feeble and exhausted. His experiments were characterized by great research and great mechanical ingenuity, and he was the first operator to apply the measure for the saving of life in cases of post-partum hemorrhage. He resorted to the operation in six cases of post-partum hemorrhage, and for a time his patients rallied.

There was a marked improvement in the action of the heart and respiration, but all of his cases terminated fatally. As far as I have been able to learn, the first case in which the operation was resorted to in connection with post-partum hemorrhage, in which the life of the patient was saved, was a case of Mr. Waller in the year 1859. His patient had suffered in the extreme from hemorrhage, when about ℥ ix. of blood taken from the arm of a healthy man were injected into the veins of his dying patient, and was followed by complete recovery.

In all these early cases the transfusion was indirect. The blood was first taken from the system of one person, drawn into a syringe, and then thrown into a vein of the person upon whom the operation was to be performed. At this time the subject had been taken up and examined with great zeal and industry by various German physiologists, and the results of their experiments seemed to prove that the cause of death in these cases was from coagulation of blood. It is to be remembered in this connection that at the time of those investigations the knowledge which we now have relative to thrombosis and embolism did not exist.

While the principle was recognized that blood could coagulate within the vessels, the true explanation was not understood, and different terms were employed to express the idea. In 1860 Prof. Martin operated upon one case, and succeeded in restoring the life of the patient. In 1861 he published a tabulated statement of 57 cases in which transfusion had been performed, and in which recovery took place in 45 cases. In all these cases transfusion was resorted to on account of post-partum hemorrhage. In connection with the fact that danger arose from coagulated blood, it was suggested that perhaps the operation would be rendered more safe if the blood was defibrinated; consequently subsequent experimenters were in the practice of defibrinating the blood by whipping it with a thin rod.

In 1860 Prof. Nusbaum performed the operation in connection with a case of disease of the knee-joint. His patient was so exhausted by the disease that resection of the joint could not be performed. Transfusion was resorted to, and the strength of the patient was re-established to such an extent that the operation was subsequently performed, and was followed by complete recovery.

The same year Prof. Esmarch resorted to transfusion in a case where profuse hemorrhage had followed amputation of the leg. He made use of calf blood, but the operation was unsuccessful.

In the same year Dr. Flint, Jr. performed the operation in the city of New Orleans, but the details of the case the Doctor, being present, will himself relate.

In 1862 ——— of Stuttgart resorted to the operation in a case of exhaustion dependent upon an alarming hemorrhage in a case of abortion, and the woman was restored to complete recovery.

In 1864, Graily Hewitt proposed an apparatus for performing the operation, which consisted of a syringe so arranged that blood could be received into the open mouth of the instrument by means of a needle something like the ordinary hypodermic needle, and through this needle the blood was to be introduced into the vein.

Dr. B. W. Richardson proposed that gravitation alone should be the medium by which the blood should be transferred to the patient. To secure this the blood was to be drawn directly into a basin, which was to be elevated some inches above the level of the body of the patient. In that manner, he claimed, the blood would be transferred slowly, and the tendency to coagulation prevented. He also urged that, in case a syringe was used for the transfer of the blood, the blood should first be whipped.

In 1868 J. Braxton Hicks, in Guy's Hospital Reports, mentions six cases in which the operation was performed, and in all the operations was unsuccessful.

He proposed, in order to prevent the tendency to coagulation, to mix with the blood a solution of phosphate of soda. Three grains of the soda were to be added to a pint of water, and then one part of the solution to three of blood was employed. In four of his cases the operation was followed by a temporary rallying of the patients, but death seemed to be produced by certain organic causes. So far as the operation of transfusion was concerned, Hicks regarded these cases as being successful, although all six of his patients died.

In 1871 Belina reported seven cases in which the operation was performed in connection with flooding after abortion. Defibrinated blood was used, and all his cases recovered.

Dr. Higginson, of Liverpool, has reported thirteen cases in which the operation was performed, ten of which were in obstetric practice. The blood used was not defibrinated, and four recoveries followed the operation.

Dr. Ringland, of Dublin, has also reported one successful case.

In this city, to my knowledge, the operation has been performed six times, but in no case has it been successful.

In the first case the operation was performed for the restoration of a woman who had fallen into a condition of extreme exhaustion incident to hemorrhage following the removal of a polypus. Her kidneys were in an advanced stage of Bright's disease.

The following notes of the case have been furnished me by Dr. F. J. Metcalf:

"Not having any regular apparatus for the operation, the house physician had to improvise as well as possible. A ligature in the form of a bandage having been placed above and below the vein, at the bend of the left elbow, the vein was opened, and the end of a fine nozzle, fitting to a three-ounce syringe, was introduced and made fast by a ligature to the vein. The median basilic of one of the doctors present having been opened and seven ounces of blood drawn, this was beaten up in a vessel with a thin whip, for the purpose of keeping it from coagulating, and was then injected into the left median basilic of the patient. In this way thirteen ounces of blood were injected. At the beginning of the operation the pulse was 130, small and feeble, temperature much fallen, skin cold and clammy, respiration 15, and woman comatose. Whilst the blood was being transfused the pulse fell to 100 or 102, and grew fuller and stronger, respiration more forcible, and the body became warmer. As soon as the syringe was withdrawn to be refilled, the pulse went up. After all the blood had been injected, the patient was given a teaspoonful of brandy and ℳ v. of tinct. digitalis every ten minutes for an hour. This had its beneficial effects for some six or eight hours, but the patient finally died at the end of forty-eight or sixty hours after the first operation."

The second case was one of post-partum hemorrhage. The patient rallied, but did not recover.

The third case was one of gastritis of a very severe form. Transfusion was followed by temporary benefit.

The fifth case was one in which the operation had been performed subsequent to the operation of ovariotomy. The following notes are from Prof. T. G. Thomas, who had charge of the case:

"Patient vomited for nine days after ovariotomy, and was sustained by enemata alone. At the end of that time her strength failed so completely that, as I saw death approaching from sheer exhaustion, I injected into the median basilic vein ℥ viii. of defibrinated human blood with a large vulcanite syringe. No sensible effect was produced, and death occurred a short time after the operation. Fifteen years ago, says the Doctor, I saw Dr. Van Buren perform the same operation upon a patient who had vomited blood to complete exhaustion. No good result followed the operation, and the patient died soon after. Post-mortem revealed large fatty liver, with open vessels over surface of stomach. Patient a drunkard. Autopsy performed by Dr. C. E. Isaacs."

The conditions which indicate the operation of transfusion are such as are obtained after hemorrhages, and such as are obtained in connection with anæmia, cholera, leuoocythemia, pyæmia, vomiting of pregnancy, etc. The number of cases of recovery, after resorting to the operation, is seventy-seven, which is sufficient to warrant a more general adoption in practice than it now receives.

Within the past two years several successful cases have been reported in England, in which the patient had been brought to very extreme exhaustion from loss of blood. Some of these cases have occurred after abortions, and some of them have occurred at full term; and the blood has been transfused directly from the doner to the receiver by means of an instrument which has been devised by Dr. Aveling, physician to Chelsea Hospital for Diseases of Women. The success of this operation, like the success of very many other great operations, will be just in proportion to the precautions of all the details and perfect familiarity with every step of the operation, in order that it may be performed with dexterity, ease, and safety to the patient.

Assistance for fulfilling these indications, it is believed, is better afforded by the instrument of Dr. Aveling than by any which has yet been devised.

Dr. Peaslee remarked that he had been struck with the fact that the use of so small a quantity of blood had caused the recovery of the patient in many reported cases—some cases where, perhaps, no more than ℥ ii. or ℥ iv. of blood had been injected, and yet the cases her terminated in recovery. He had been inclined to reject these individual cases as certainly not being very reliable; but there are cases which prove transfusion, without any chance of contradiction, has saved the life of the patient. It is probable that many cases in which the operation has been tried have not been fever cases, and it is to be hoped that such an instrument as that invented by Dr. Aveling may prove safe and reliable.

Dr. Barker desired to call attention somewhat more definitely to cases of excessive exhaustion resulting from the sickness of pregnancy. The propriety of producing premature labor in certain cases is now pretty well settled; still, patients do fall under observation in which there is so much exhaustion that it would be folly to resort to the procedure in actual fear that death may thereby be produced. It is in this class of cases that the operation of transfusion would be suggested, in the hope, at last, of restoring the strength of the patients sufficiently to enable them to withstand the shock of the operation which is sometimes so imperative."—*Med. Record.*

